# Abstract of the Proceedings of the Buffalo Medical Association

**Published:** 1857-01

**Authors:** Sanford B. Hunt


ART. VII.—*Abstract of the Proceedings of the Buffalo Medical Association.*

THURSDAY EVENING, Dec. 2, 1856.

The association met.

Owing to the omission of the usual notice, but a small number of members were present.

*Prof. Hamilton* asked permission of the association to introduce Dr. McLean, of Niagara county, who had a new and valuable cupping apparatus which he wished to show to members. It had previously been exhibited to the State Medical Society. Dr. McLean was a good and worthy practitioner, and he moved that he be invited to address the association. *Motion carried.*

Dr. McLean's apparatus consists of several large disks of brass connected with an air-pump. The pump had the ordinary puppet-valve, but it was held in place by a coil of wire, so that it was not necessary to keep the pump in a perpendicular position when in use. The cups, seven in number, varied in size from the ordinary small cup for the temples, to one seven inches in diameter, to be applied over the bowels. Each cup had an inner disk which divided it into two which were connected by air holes. The object of this was to prevent too great distension of the skin, and thus divide and equalize the pressure.

Dr. McLean said he had a practical knowledge of the therapeutic effects of this cup, having used it somewhat largely. The smaller cups did not differ from those previously in use except in greater convenience and beauty, the larger ones went beyond the principal of counter-irritation, or mere local abstraction of blood, and were intended to control the general circulation.

They had been somewhat largely used by Dr. Sanger, at Blackwell's Island. In dysentery he had himself used them with surprising effect. The large amount of blood drawn with the largest cups, had as much effect as a general bloodletting, without making any deduction from the circulating fluid. He had never used it in uterine hæmorrhage. In dysentery the tormina subsided immediately under its use, and convalescence soon followed. In sciatica he had once applied a large cup over the hip-joint for relief of the pain for an hour. It produced almost an entire vesication, with immediate and permanent relief to the neuralgic difficulty. In spinal irritation, congestion of the lungs, and in rheumatism, he had found it very efficacious.

Dr. McLean then applied a six inch cup to himself for a few moments. It produced very considerable irritation. The large cup may produce syncope. The price of the entire instrument is only $15.

*Prof. Hamilton* exhibited a case of "*Exostosis*" *of a Tooth.*

Specimen of "*exostosis*" *of a tooth*, presented to me by Charles W. Harvey, M. D., of this city. The patient, a gentleman about — years of age, had suffered for many years from what had been supposed to be neuralgia, which finally produced insanity. Under these circumstances he was brought to Dr. Harvey for the purpose of having one of his teeth extracted.

With great difficulty, and only after applying extraordinary force, he removed this tooth. The tooth was found to be sound, but there is seen attached to it a growing from its roots, near the crown, a round, smooth, solid tumor of bone, of about the size of a small filbert. This mass resembles the structure of the root from which it seemed to have grown, but along its upper enamel, and which seems to have grown from it, and to resemble it in structure.

No accident followed the operation. The neuralgia immediately ceased, and Dr. Harvey understands that the patient was soon restored to sanity.

*Prof. Hamilton* stated, in reply to inquiry, that the case of *lupus non exedens*, exhibited by him at a previous meeting, had entirely recovered. The patient took fifteen drops of Fowler's solution, three times daily, for a fortnight, then suspended the medicine for a week, and then resumed in smaller doses. The result had been, as before stated, a perfect cure. He regarded the dose as very large, but the case was desperate and spreading very rapidly.

*Prof. Rochester* reported a case of psoriasis, which, when he first saw it, extended between the shoulders over the back part of the neck over one side of the face and the whole forehead, stopping at the roots of the hair. The woman had had it a year. The margin of the eruption was, throughout, very distinct. There was no derangement of the general health. She was ordered to take Fowler's solution, in five drop doses, three times daily, increasing one drop each day. At the end of fourteen days the cure was complete.

*Dr. Newman* mentioned the excessive caution urged by Rayer in using Fowler's solution. It would seem, from the doses given by Prof. Hamilton, that Rayer was unnecessarily careful.

*Dr. Dayton* said he had been in the habit of giving from ten to fifteen drops at a dose, in intermittent fever.

*Dr. Newman* described a case of lepra on the back of a child. As the child had been exposed to small-pox he vaccinated it, and a few weeks after learned that the lepra had disappeared during the eruptive fever of the vaccinia.

*Prof. Rochester* said that vaccinia frequently exerted an important influence over eruptive diseases.

In another connection he mentioned the case of a child whom he vaccinated simultaneously with two others, from the same virus. The child was immediately taken to Fredonia, from whence he received a letter stating that a variolous eruption had followed the insertion of the virus, and that Prof. McNaughton, of Albany, had seen it, and declared that the virus must have been impure. Prof. Rochester did not believe that Prof. McNaughton could have been guilty of so great an impropriety as uttering such a censure in a case of which he could have no accurate knowledge. The two other children vaccinated at the same time, passed through the usual phenomena of vaccinia. Prof. R. stated that he had known several instances of pustular eruption of irregular kind, to follow vaccination. This was probably one of them.

*Dr. Dayton* proposed Dr. Augustus Jeyte for membership.

And the association then adjourned.

SANFORD B. HUNT, Secretary.

